# Regulation of interleukin-1 beta gene expression and its function of defense mechanism in rumen epithelial cells from pre- and postweaning calves

**DOI:** 10.5713/ab.25.0042

**Published:** 2025-05-19

**Authors:** Huseong Lee, Naoto Sugiyama, Koki Nishihara, Minji Kim, Satoshi Haga, Sanggun Roh

**Affiliations:** 1 Graduate School of Agricultural Science, Tohoku University, Sendai, Japan

**Keywords:** Chemokine, Flagellin, Lipopolysaccharide, Rumen Epithelium, Short-chain Fatty Acids

## Abstract

**Objective:**

This study aimed to elucidate interleukin-1 beta (IL-1β) gene expression regulation and its function of defense mechanism in rumen epithelial cells of calves.

**Methods:**

Rumen tissues from six Holstein male calves were sampled at pre- (5 weeks of age, n = 3) and post-weaning (9 weeks of age, n = 3). IL-1β localization was analyzed using immunohistochemistry (IHC). Primary bovine rumen epithelial cells (BRECs) were treated with short-chain fatty acids (SCFAs), beta-hydroxybutyrate, lactic acid, lipopolysaccharide (LPS), and flagellin, and IL-1β gene expression was analyzed by quantitative real-time polymerase chain reaction. Additionally, bovine IL-1β-treated BRECs were assessed for cell proliferation, tight junction (TJ) protein expression, and chemokine mRNA expression.

**Results:**

IHC revealed IL-1β expression across all rumen epithelial layers. SCFAs, LPS, and flagellin significantly increased IL-1β mRNA expression (p<0.05). Regarding the gene expression of rumen TJ proteins, *CLDN4* and *OCLN* in suckling and weaned calves, as well as ZO-1 in weaned calves, showed significant decreases (p<0.05), while *CLDN1* in weaned calves showed a significant increase (p<0.05) in IL-1β-treated BRECs. Regarding the gene expression of chemokines, the *CCL2* expression significantly decreased (p<0.05), while the *CCL5* expression significantly increased (p<0.05) in both suckling and weaned IL-1β treated BRECs. IL-1β treatment enhanced cell proliferation (p<0.05).

**Conclusion:**

These results suggest that IL-1β induction by SCFAs, LPS, and flagellin, which increase in the rumen due to environmental changes, promotes cell proliferation in damaged rumen epithelium and contributes to its defense mechanism by upregulating *CCL5* expression.

## INTRODUCTION

The rumen is a vital digestive organ in cattles, performing several essential physiological functions, including homeostasis and the uptake, transport, and metabolism of nutrients [1]. The rumen is underdeveloped at birth but grows rapidly during the weaning period [2]. This development is critical during the transition from milk to solid diets in calves [3], with short-chain fatty acids (SCFAs) produced by rumen microbes becoming the primary energy source for mature cattles. These SCFAs, that include acetate, butyrate, and propionate, promote rumen papillae growth and enhance SCFAs absorption efficiency [4,5]. Thus, SCFA production is closely associated with rumen development during the weaning period.

However, in dairy calf management, weaning increased intake of readily fermentable carbohydrates, increasing SCFA production and lowering rumen pH [6]. Both acute and subacute ruminal acidosis can disrupt rumen barrier, leading to inflammation and negatively affecting the health of calves [7]. The rumen epithelium consists of stratified squamous epithelium with tight junctions (TJs). These TJs is essential for maintaining health and preventing damages from ruminal acidosis, particularly in ruminants fed high grain diets [8]. Understanding the adaptation and defense mechanism of rumen epithelial cells to environmental changes and their role in maintaining homeostasis during weaning period could help develop strategies for preventing or alleviating ruminal acidosis and secondary inflammation.

Interleukin-1 beta (IL-1β) is a potent pro-inflammatory cytokine and a key member of the IL-1 family. It acts as a mediator in homeostatic functions and host-defense responses to infection and injury [9]. In human skin, IL-1β increases rapidly after tissue injury, peaking within 12 to 24 h, and return to basal levels once the proliferation phase of wound healing concludes [10]. IL-1β also promotes early recruitment of neutrophils to the injury site, stimulates the proliferation of fibroblasts and keratinocytes, and enhances the production of extracellular matrix proteins, including collagen, elastin, fibronectin, and laminin [11,12]. IL-1β strengthens barriers in human gingival epithelial cells, promotes cell proliferation in rat dental pulp cells [13,14], and influences antimicrobial peptide expression and neutrophil accumulation in bovine oviduct epithelial cells [15]. The role of IL-1β on barrier and inflammation functions in the rumen epithelium of calves during the weaning period has not been extensively studied. Therefore, investigating the physiological role of IL-1β in the rumen epithelium during weaning could be beneficial for rumen development, health, and growth improvement in calves.

Therefore, in this study, we aimed to elucidate the role of IL-1β in the rumen epithelium by examining rumen epithelial cell adaptation to environmental changes and homeostasis maintenance. Specifically, we investigated the regulation of IL-1β gene expression in cultured rumen epithelial cells of calves around weaning and explored the effects of IL-1β on cell proliferation and expression of ruminal TJ and chemokine genes.

## MATERIALS AND METHODS

### Animals

This study was conducted according to the “Guideline for the Tohoku University” and approved by the Animal Care Committee of Tohoku University (2016AgA-004). Six Holstein male calves, bred at the Graduate School of Agricultural Science, Tohoku University, Sendai, Japan, were randomly assigned to two experimental groups: suckling (n = 3) and weaned (n = 3). Holstein male calves were chosen due to their genetic uniformity, which minimizes individual variation in cellular responses, as demonstrated in our previous study [16,17]. The comparison between pre- and post-weaning stages was intended to capture dynamic changes in rumen development and immune responses during this transitional period. All calves were separated from dams following colostrum intake and provided with a milk replacer. The calves were fed a milk replacer (Calftop EX; Zenrakuren, Tokyo, Japan) twice daily, at 09:00 h and 16:00 h. Calves in the suckling group were exclusively raised on artificial suckling without a feeding starter and slaughtered at 5 weeks of age. Calves in the weaned group were introduced to a calf starter (New make star, Zenrakuren) at 1 week of age, weaned at 7 weeks, and slaughtered at 9 weeks of age. All calves had *ad libitum* access to water. The nutritional composition and feeding amounts of milk replacer and starter are detailed in Supplement 1.

### Tissue collection

The calves were euthanized by exsanguination after an overdose of anesthetic (thiopental sodium, Ravonal, NIPRO ES Pharma, Osaka, Japan), which induced complete loss of consciousness. This method was selected because thiopental sodium induces rapid and deep anesthesia, minimizing pain and distress prior to exsanguination. The procedure was conducted in accordance with the animal care guidelines and approved protocol mentioned above, ensuring humane treatment. Immediately after slaughter, rumen papillae tissues (approximately 1×1 cm) were collected from the ventral cranial sac, an anatomically well-defined region characterized by prominent papillary development and high sensitivity to fermentative and absorptive changes. The papillae layer was carefully separated from the muscular layer using surgical scissors and washed with phosphate-buffered saline (PBS) to eliminate any remaining feed particles. The tissue samples were immediately frozen in liquid nitrogen and stored at −80°C until further use. Samples intended for immunohistochemistry were fixed in Bouin’s solution and subsequently embedded in paraffin.

### Immunohistochemical assay

Sectioned rumen papillae samples (3 μm thickness) were deparaffinized in xylene and then rehydrated using alcohol. Then, the sections were subjected to antigen retrieval by autoclaving at 120°C for 10 min in 0.01 M citric acid buffer (pH 6.0), followed by cooling to 25°C. After washing in PBS containing Tween 20 (PBST), the tissue sections were blocked using a buffer containing 5% normal goat serum diluted in 1× PBST and incubated overnight at 4°C with rabbit anti-bovine IL-1β IgG (1:1,000; KP1109B-100; Kingfisher Biotech, St Paul, MN, USA) as the primary antibody. After a second wash in PBST, the sections were incubated with the Histofine Simple Stain MAX-PO (R) (goat anti-rabbit IgG) detection reagent (424141; Nichirei Bioscience, Tokyo, Japan). Following a final wash with PBST, the localization of IL-1β was visualized using a DAB substrate kit (425011; Nichirei Bioscience). The sections were counter-stained with hematoxylin for 1 min. Subsequently, the tissue sections were dehydrated using alcohol and xylene, and mounted using Softmount (FUJIFILM Wako Pure Chemical, Osaka, Japan). Tissue sections where the primary antibody was omitted were used as negative controls. All sections were examined using an Olympus BX50 microscope (Olympus, Tokyo, Japan).

### Cell isolation and culture

Rumen tissues collected from the ventral cranial sac were immersed in ice cold PBS containing 1% penicillin-streptomycin (PS-PBS; 168-23191; FUJIFILM Wako Pure Chemical) until cell isolation. The muscle layers were removed before isolating the cells. Rumen papillae were collected in 50 mL centrifuge tubes and washed 10 times with 15 mL of PS-PBS. Then, the papillae were treated with 0.25% trypsin (201-16945; FUJIFILM Wako Pure Chemical) containing 2 U/mL deoxyribonuclease (313-03161; NIPPON GENE, Tokyo, Japan) for 30 min at 37°C to remove the corneum layer. After three washes with PS-PBS, the papillae were transferred to 0.25% trypsin kept on ice and minced with surgical scissors. The minced tissue was incubated with 0.25% trypsin at 37°C for 30 min, then strained through a 70 μm cell strainer. The filtrate was mixed with high-glucose Dulbecco’s Modified Eagle Medium (DMEM; 043-30085; FUJIFILM Wako Pure Chemical) containing 10% fetal bovine serum (FBS; Lot No. 11464, FB-1061/500; Biosera, Sussex, UK) at a 1:1 (v/v) ratio and centrifuged at 160×g for 5 min at 4°C. After aspirating the supernatant, the cell pellet was resuspended in high-glucose DMEM containing 10% FBS, 1% penicillin-streptomycin, 20 mM 4-(2-hydroxyethyl)-1-piperazineethanesulfonic acid (HEPES), 1 nM insulin (I9278, Sigma-Aldrich, St. Louis, MO, USA), and 1 μM dexamethasone (D4902; Sigma-Aldrich). Cell counts were determined using a Countess II FL cell counter (Thermo Fisher Scientific, Waltham, MA, USA). Primary bovine rumen epithelial cells (BRECs) were seeded at 1.0×10^6^ cells/well in a 6-well plate and incubated at 37°C in 5% CO_2_ in a humidified incubator, with medium changes every 24 h. After 6–7 days of culture (confluency>80%), the BRECs were incubated without insulin and dexamethasone for 24 h before further use.

### Cell stimulation and quantitative real-time polymerase chain reaction

To evaluate the effects of SCFAs concentrations on IL-1β expression, the BRECs were cultured in a medium at pH 7.4 (physiologically normal intracellular pH) either without Na-SCFA (control) or with 100 mM Na-SCFA, composed of 60 mM sodium acetate (191-13912; FUJIFILM Wako Pure Chemical), 30 mM sodium propionate (37253-00; Kanto Kagaku, Tokyo, Japan), or 10 mM sodium butyrate (193-01522; FUJIFILM Wako Pure Chemical) for 3 h. The pH of the medium was adjusted using gluconic acid (G0036; Tokyo Kasei, Tokyo, Japan) and 100 mM Na-gluconate (G0041; Tokyo Kasei) in the Na-SCFA-free medium. For investigating the effect of beta-hydroxybutyrate (BHBA) on IL-1β expression, the BRECs were incubated in a medium (pH 7.4) containing 10 mM Na-BHBA (085-03571; FUJIFILM Wako Pure Chemical) for 3 h. To examine the impact of L-lactate and D-lactate on IL-1β expression, the BRECs were treated with 1 mM L-lactic acid (L0165; Tokyo Kasei) or 1 mM D-lactic acid (L0266; Tokyo Kasei) for 3 h. The control group’s medium contained 10 mM Na-gluconate, while that of the L-lactate and D-lactate groups contained 9 mM Na-gluconate. To assess the effect of lipopolysaccharide (LPS) on IL-1β expression, the BRECs were exposed to 5,000 endotoxin units/mL of LPS from *Escherichia coli* O55:B5 (L2880; Sigma-Aldrich) for 24 h. To determine the impact of bacterial flagellin on IL-1β expression, the BRECs were cultured in a medium containing water-dissolved flagellin (ALX-522-058; Enzo Life Sciences, Farmingdale, NY, USA) at final concentrations of 10 or 100 ng/mL for 24 h. The stimulant concentrations were within the physiological range, and both the concentrations and incubation times were based on our previous study [18].

To investigate the effect of IL-1β on the expression of genes encoding ruminal TJ proteins, including claudin-1 (*CLDN1*), claudin-4 (*CLDN4*), claudin-7 (*CLDN7*), occludin (*OCLN*), and zonula occludens-1 (ZO-1), as well as chemokines, including C-C motif chemokine ligand 2 (*CCL2*) and C-C motif chemokine ligand 5 (*CCL5*), the BRECs were treated with 0.1 or 1.0 ng/mL recombinant bovine IL-1β (RP0106B-005; Kingfisher Biotech) for 24 hours.

Following the treatments, the BRECs were washed with PBS, and total RNA was isolated using RNAiso (TaKaRa-Bio, Shiga, Japan). Subsequently, the total RNA was reverse transcribed using the PrimeScript™ RT reagent kit with gDNA Eraser (Perfect Real Time; TaKaRa-Bio). Gene expression was analyzed using quantitative real-time polymerase chain reaction (qRT-PCR) with SYBR Premix ExTaq II (Tli RNaseH Plus; TaKaRa-Bio). Primer sequences are listed in Supplement 2. Beta actin and 18S mRNA were used as reference genes. Post-PCR melting curves were observed to confirm the specificity of single-target amplification. Relative mRNA expression was calculated using the modified 2^-ΔΔCt^ method [19].

### 3-(4,5-Dimethylthiazol-2-yl)-2,5-diphenyltetrazolium bromide assay

The BRECs isolated from suckling calves (suckling BRECs) were incubated for 24 h in serum-starvation medium (DMEM with 0.1% bovine serum albumin [A9647; Sigma-Aldrich], 1% penicillin-streptomycin, 20 mM HEPES) (control) or in serum-starvation medium supplemented with 0.5, 1, or 5 ng/mL recombinant bovine IL-1β (Kingfisher Biotech). IL-1β was dissolved in serum starvation medium. After 24 h, cell viability was assessed using the 3-(4,5-dimethylthiazol-2-yl)-2,5-diphenyltetrazolium bromide (MTT) cell viability assay. Briefly, 10 μL of the MTT solution (298-93-1; Sigma-Aldrich) was added to 100 μL of culture medium. After 2 h of incubation at 37°C, 100 μL of hydrogen chloride in 2-propanol solution was added to each well. Absorbance was measured at 550 nm using the Synergy HT multi-mode microplate reader (BioTek Instruments, Winooski, VT, USA).

### Statistical analyses

Data are presented as mean ± standard error of the mean from experiments conducted in triplicate dishes for each animal to ensure technical reproducibility and minimize intra-animal variability. Student’s t-test was used to assess differences in IL-1β expression in the BRECs following SCFA, BHBA, and LPS stimulation, as each comparison involved two independent groups (control vs. treated) and satisfied the assumptions of normality and equal variance. One-way analysis of variance with Tukey’s HSD test was used to compare IL-1β expression in BRECs after L-lactate, D-lactate and flagellin stimulation, as well as mRNA levels of ruminal TJ and chemokine genes and cell proliferation rates following IL-1β treatment. Statistical analyses were performed using R version 3.6.2 (https://www.r-project.org) with significance set at p<0.05.

## RESULTS

### Localization of interleukin-1 beta in the rumen papillae

**Figure 1:**
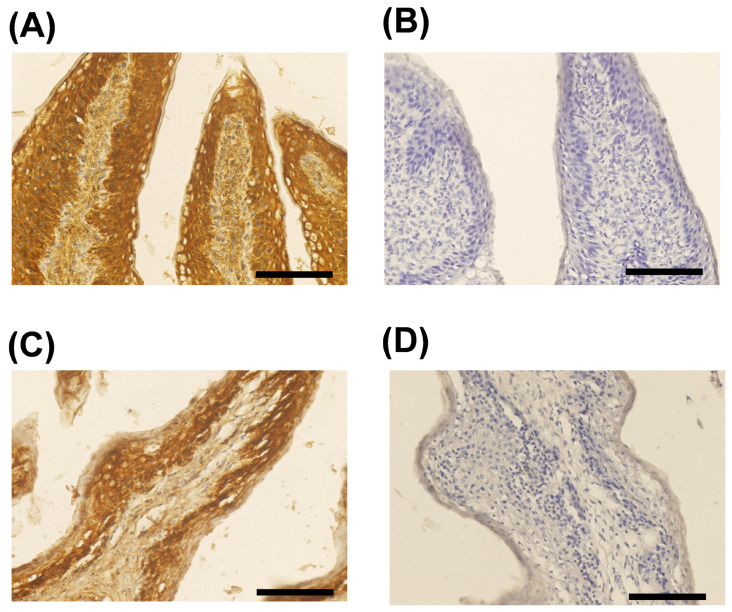
Localization of IL-1β in rumen papillae of suckling and weaned calves. (A) Rumen papillae of suckling calves (5 weeks of age). (B) Rumen papillae of suckling calve without the first antibody as the negative control. (C) Rumen papillae of weaned calve (9 weeks of age). (D) Rumen papillae of weaned calve without the first antibody as the negative control. IL-1β: brown color; Nuclear: blue color (Hematoxylin); scale bar = 500 μm. IL-1β, interleukin-1 beta.

Figure 1 illustrates the localization of IL-1β in the rumen papillae of suckling and weaned calves. For both groups, IL-1β was present throughout the entire rumen epithelial layer, particularly localized in the granular, basal and spinous layers (Figures 1A, 1C).

### Effect of short-chain fatty acids, beta-hydroxybutyrate, L-lactate, D-lactate, lipopolysaccharide, and flagellin treatment on interleukin-1 beta gene expression in bovine rumen epithelial cells

**Figure 2:**
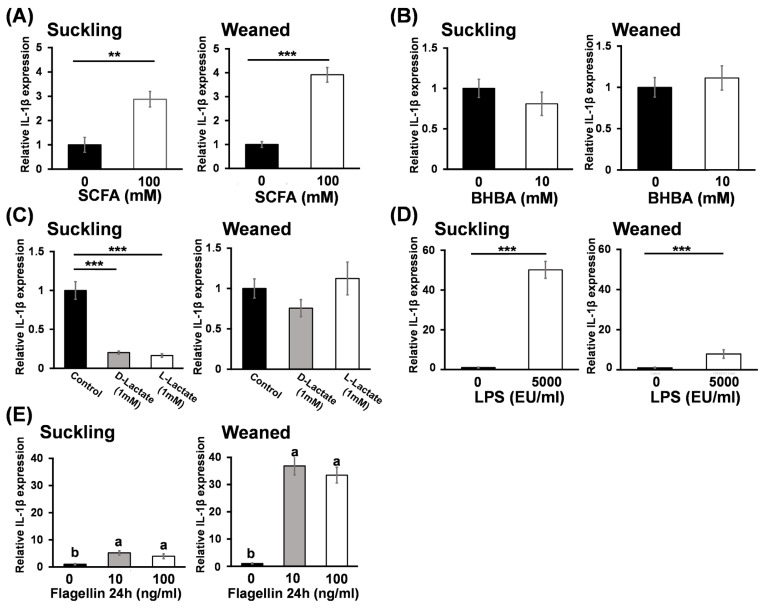
The effects of SCFA (A), BHBA (B), D-lactic acid, L-lactic acid (C), LPS (D) and Flagellin (E) on expression of IL-1β mRNA in suckling and weaned BREC. The relative mRNA expression levels were analyzed using qRT-PCR and are presented as mean±standard error of the mean relative to the control, which was normalized to 1. Statistical significance was indicated as ** for p<0.01 and *** for p<0.001 compared to the control (A, B, C, and D; Student’s t-test). ^a,b^ Different letters represent significant differences between groups (p<0.05, Tukey’s HSD test). SCFA, short-chain fatty acid; BHBA, beta-hydroxybutyrate; LPS, lipopolysaccharide; IL-1β, interleukin-1 beta; BREC, bovine rumen epithelial cell; qRT-PCR, quantitative real-time polymerase chain reaction.

The effects of SCFAs, BHBA, L-lactic acid, D-lactate, LPS, and flagellin treatment on IL-1β mRNA expression in BRECs from suckling and weaned calves are shown in Figure 2. SCFAs treatment significantly increased IL-1β mRNA expression levels for both groups compared to the control (p<0.01; Figure 2A). However, BHBA did not affect IL-1β gene expression. L-lactate and D-lactate did not alter IL-1β mRNA expression levels in BRECs from weaned calves (weaned BRECs), but significantly downregulated IL-1β mRNA expression in suckling BRECs (p<0.01; Figure 2C). Furthermore, LPS and flagellin treatments significantly increased IL-1β mRNA expression levels in BRECs compared to the control groups (p<0.001 and p<0.05, respectively; Figures 2D, 2E). Notably, LPS increased IL-1β mRNA expression by approximately 8.14-fold in suckling BRECs compared to the weaned BRECs, while flagellin increased IL-1β mRNA expression by approximately 7.69-fold in weaned BRECs compared to suckling BRECs.

### Effect of interleukin-1 beta on the expression of genes encoding ruminal tight junction proteins and chemokines in bovine rumen epithelial cells

**Figure 3:**
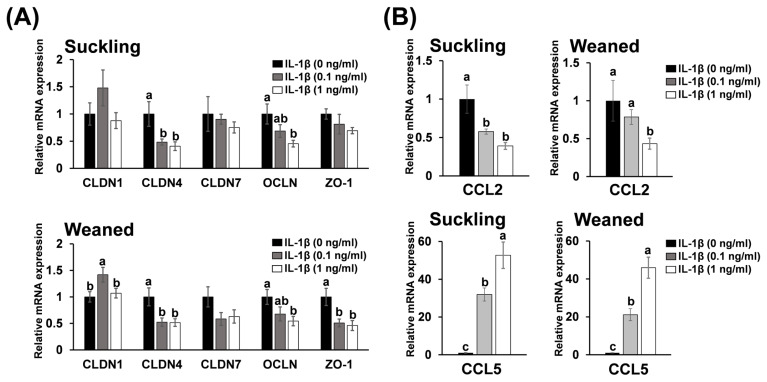
The effect of IL-1β on gene expression of tight junction (TJ) proteins (A) and chemokines (B) in suckling and weaned BREC. The relative mRNA expression levels were analyzed using qRT-PCR and are presented as mean±standard error of the mean. We set the value at 0 ng/mL of IL-1β as the reference value, which was normalized to 1. ^a–c^ Different letters represent significant differences between groups (p<0.05, Tukey’s HSD test). IL-1β, interleukin-1 beta; BREC, bovine rumen epithelial cell; qRT-PCR, quantitative real-time polymerase chain reaction.

Figure 3 shows the effects of IL-1β on the expression of genes encoding TJ proteins and chemokines in suckling and weaned BRECs. *CLDN4* expression in both groups of BRECs treated with 0.1 or 1.0 ng/mL IL-1β, *OCLN* expression in both groups of BRECs treated with 1.0 ng/mL IL-1β, and *ZO-1* expression in the weaned BRECs treated with 0.1 or 1.0 ng/mL IL-1β was significantly lower than that in the control BRECs (p<0.05; Figure 3A). In contrast, *CLDN1* expression in weaned BRECs treated with 0.1 ng/mL IL-1β was significantly higher than that in control BRECs (p<0.05; Figure 3A). Additionally, CCL2 expression in the suckling BRECs treated with 0.1 or 1.0 ng/mL IL-1β and in the weaned BRECs treated with 1.0 ng/mL IL-1β was significantly lower than that in the control BRECs (p<0.05; Figure 3B). In contrast, *CCL5* expression in both groups of BRECs treated with 0.1 or 1.0 ng/mL IL-1β was significantly higher than that in the control BRECs (p<0.05; Figure 3B).

### Effect of interleukin-1 beta on bovine rumen epithelial cell proliferation

**Figure 4:**
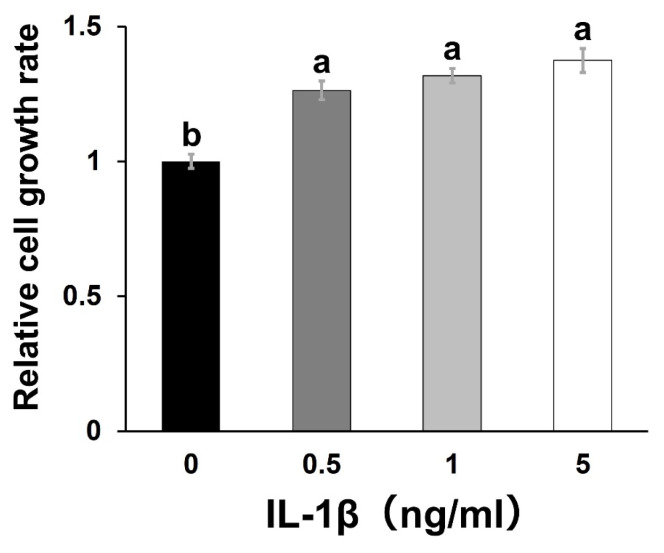
The effect of IL-1β on cell proliferation in suckling BREC. Relative cell growth rate was analyzed by MTT assay and are shown as mean±standard error of the mean. We set the value at 0 ng/mL of IL-1β as the reference value. ^a,b^ Different letters indicate significant differences between groups (p<0.05, Tukey’s HSD test). IL-1β, interleukin-1 beta; BREC, bovine rumen epithelial cell; MTT, 3-(4,5-dimethylthiazol-2-yl)-2,5-diphenyltetrazolium bromide.

Treatment with IL-1β (0.5, 1, or 5 ng/mL) significantly increased the proliferation rate of suckling BRECs compared to the control group (0 ng/mL) (p<0.05; Figure 4).

## DISCUSSION

This study is the first to investigate the direct impact of ruminal metabolites on IL-1β gene expression and to elucidate the role of IL-1β on barrier and inflammation functions in the BRECs from calves around weaning. Both suckling and weaned BRECs exhibited similar cellular responses, with minimal phenotypic differences attributed to weaning. This is likely due to incomplete development of the rumen at these stages.

We investigated factors involved in the regulation of IL-1β gene expression and evaluated the effects of IL-1β on the expression of ruminal TJ and chemokine genes in cultured BRECs. Considering that IL-1β plays a key role in wound healing and barrier function in other stratified squamous epithelia, such as the skin, we hypothesized that it may have a similar role in the rumen epithelium. The findings showed that SCFAs, LPS, and flagellin upregulated IL-1β expression. IL-1β promoted cell proliferation and upregulated CCL5 expression while downregulating the expression of genes encoding certain TJ proteins. SCFAs, particularly butyrate, are important fermentation byproducts in the rumen that serve as energy sources and help maintain the integrity of the epithelial barrier. They also modulate cellular functions, including proliferation and inflammation regulation [20]. SCFAs regulate GPR41-mediated expression of IL-1β, promoting protective innate immunity in BRECs of 6- to 7-month-old Holstein calves [21]. In the current study, treatment with SCFAs significantly increased IL-1β expression in both suckling and weaned BRECs compared to the controls, suggesting that SCFAs may regulate the defense response mediated by IL-1β in the rumen epithelium during early life as well. Feeding easily fermentable diets can lead to the production of D-lactic acid and L-lactic acid due to rapid carbohydrate fermentation in the rumen [22]. The accumulation of these organic acids lowers ruminal pH and contributes to severe ruminal acidosis, which can damage rumen epithelial cells and inhibit their proliferation [23]. Early weaned calves showed lower ruminal pH and higher SCFAs and lactate concentrations due to the higher starter intake compared to calves in the conventional weaning program [24]. In the current study, both D-lactate and L-lactate down-regulated IL-1β expression in suckling BRECs. This lactate-induced damage may have been more pronounced in suckling calves due to their underdeveloped rumen epithelium, and the defense response of the ruminal TJs, mediated by IL-1β, may have been insufficient to mitigate the damage.

Translocation of PAMPs, such as LPS, across the rumen epithelium, occurring due to acidosis and hyperosmotic conditions, can weaken the epithelial barrier [25]. This translocation can trigger a local innate immune response via pattern recognition receptors, including TLRs [26]. LPS binds to TLR4 receptor, activating the NF-κB pathway and inducing the secretion of inflammatory cytokines such as TNF-α, IL-6, and IL-1β in BREC [27]. Our study demonstrated that LPS treatment for 24 h significantly upregulated IL-1β expression in both suckling and weaned BRECs, suggesting that LPS may induce IL-1β expression through TLR4 signaling. Repeated or prolonged exposure to LPS dampened proinflammatory gene expression, such as TLR4, TNF, and IL7, indicating the presence of LPS tolerance in BRECs [28]. In our study, suckling BRECs exhibited higher IL-1β expression compared to weaned BRECs, suggesting that weaned BRECs may have LPS tolerance, possibly due to their higher grain consumption compared to suckling BRECs. Further research is needed to explore the potential effects of weaning on LPS tolerance in BRECs.

Flagellin, a protein found in bacterial flagella, triggers a proinflammatory response in epithelial tissues [29]. TLR5 is the only TLR that recognizes flagellin [30]. Weaned Japanese black and Holstein calves show higher ruminal TLR5 expression than suckling calves [31,32]. The increased expression of ruminal TLR5 related to ruminal acidosis induced by high-grain feeding or weaning and TLR5-IL-1β axis regulates the inflammatory response under these conditions [18]. Flagellin significantly upregulated IL-1β gene expression in both suckling and weaned BRECs, indicating that flagellin induces an IL-1β response even without prior exposure to weaning-related inflammation. However, weaned BRECs showed higher IL-1β expression compared to suckling BRECs, suggesting greater inflammatory responsiveness than suckling BRECs. Collectively, our results suggest that SCFAs, LPS, and flagellin can upregulate IL-1β expression in the rumen epithelium of calves, with distinct regulatory patterns observed between pre- and post-weaning periods.

The impact of IL-1β on TJs varies across different epithelial cell types. In hepatocytes, IL-1β increases OCLN expression [33]. In mammary epithelial cells, IL-1β decreases the expression of *CLDN4*, and *OCLN*, weakening the TJ barrier [34]. In the current study, IL-1β treatment reduced *CLDN4* and *OCLN* expression in both groups of BRECs and *ZO-1* expression in weaned BRECs. However, IL-1β treatment increased *CLDN1* expression in weaned BRECs, suggesting a distinct role for this protein in the rumen epithelium compared to other epithelial tissue. A high-grain diet increased IL-1β and CLDN1 gene and protein expression, while reducing CLDN4, OCLN, and ZO-1 expression in rumen epithelial cells [8,35], supporting our findings. Thus, our study confirms that increase in IL-1β in the rumen causes changes in the expression of genes encoding TJ proteins. Silencing *OCLN* in epithelial cells led to increased permeability to divalent organic cations and small molecules [36]; loss of *OCLN* in ARPE-19 cells enhanced DNA synthesis rate and cell proliferation [37]; and absence of *ZO-1* enhanced proliferation of Madin-Darby canine kidney cells and embryonic stem cells [38]. Collectively, these findings suggest that IL-1β may promote cell proliferation in rumen epithelium by down-regulating the expression of TJ proteins such as *CLDN4*, *OCLN*, and *ZO-1*. In fact, IL-1β promoted cell proliferation in BRECs. Similar effects have been observed in retinal pigment epithelial cells [39,40] and dental pulp cells through the regulation of Matrix metalloproteinase-3 [14]. This suggests that IL-1β may play a role in repairing rumen epithelial tissue by promoting cell proliferation following epithelial disruption. However, *CLDN1* does not play the same role in maintaining epithelial barriers as it does in the rumen epithelium [8], colon [41] and esophagus [42]. Unlike *CLDN4* and *OCLN*, which are primarily located in the granulosum stratum, *CLDN1* expression is more pronounced in the deeper layers of rumen epithelium [8]. A decrease in these TJ proteins could lead to an increase in *CLDN1* expression. Further research is needed to understand the mechanism behind the elevated expression of *CLDN1* in response to IL-1β stimulation in BRECs.

IL-1β induces the specific release of *CCL5* in lower airway epithelial cells through transcriptional activation. Additionally, it promotes neutrophil accumulation in oviduct epithelial cells by upregulating *CCL2* [15,43]. In the current study, IL-1β upregulated *CCL5* expression but down-regulated *CCL2* expression. Notably, while *CCL5* predominantly attracts bovine classical monocytes, *CCL2* does not exhibit the same effect [44]. Monocytes are critical immune cells that bridge innate and adaptive immunity, serving as important components of the initial defense against pathogens [45]. These findings suggest that in response to rumen tissue injury, IL-1β upregulates *CCL5* expression, promoting monocyte migration in rumen papillae. Further research is needed to explore the mechanisms underlying the down-regulation of *CCL2* expression and its implications for immune cell recruitment in the rumen.

## CONCLUSION

This study investigated the regulation of IL-1β gene expression and its function of defense mechanism as well as on the expression of genes encoding ruminal TJs and chemokines in cultured rumen epithelial cells in calves around weaning. The findings showed that SCFAs, LPS, and flagellin upregulated IL-1β expression, with differing regulatory responses between suckling and weaned BRECs. IL-1β promoted cell proliferation and upregulated *CCL5* expression while downregulating the expression of genes encoding certain ruminal TJ proteins. These results suggest that IL-1β may play a role in the proliferation phase of wound healing in the rumen epithelium and could serve as a target for preventing or alleviating rumen acidosis symptoms around weaning period.

These findings contribute to our understanding of immune responses and epithelial barrier function in the rumen and suggest that modulation of IL-1β signaling could be utilized to enhance ruminal health, support epithelial recovery, and improve the nutritional resilience of calves during the weaning transition. To build on these results, further research should investigate the molecular mechanisms regulating IL-1β expression in rumen epithelial cells and evaluate the potential of IL-1β-targeted approaches for maintaining epithelial integrity and preventing ruminal dysfunction in young calves.

## SUPPLEMENTARY MATERIAL

Supplement 1. The total amount of fed milk replacer and starter in a day.

Supplement 2. Primers used in qRT-PCR.
