# Supplementary material for: Regulation of interleukin-1 beta gene expression and its function of defense mechanism in rumen epithelial cells from pre- and postweaning calves
Source: Anim Biosci. 2025 May 19;38(11):2454–63. doi: 10.5713/ab.25.0042 (PMC12580949; doi:10.5713/ab.25.0042)
Supplement: Supplementary file 1 [file ab-25-0042-supplementary-1.pdf]

Supplement 1. The total amount of fed milk replacer and starter in a day

	Pre-weaning group		post-weaning group	
Week	Calf starter (g)	Milk replacer (g)	Calf starter (g)	Milk replacer (g)
0-1				
1-2	100	300	100	300
2-3	150	400	150	400
3-4	200	600	200	600
4-5	300	600	300	600
5-6			400	600
6-7			500	400
7-8			600	200
8-9			700	0

Milk replacer was dissolved in 1 L of warm water per 200 g and fed at 9:00 and 16:00 using a feeding bucket. Starter was also fed at 9:00 am.

Calf starter: Total digestible nutrients (TDN) >103%, crude protein (CP) >28%, crude fat >15%

Milk replacer: TDN >72.0%, CP >18.0%, crude fat >2%
