# Supplementary material for: Regulation of interleukin-1 beta gene expression and its function of defense mechanism in rumen epithelial cells from pre- and postweaning calves
Source: Anim Biosci. 2025 May 19;38(11):2454–63. doi: 10.5713/ab.25.0042 (PMC12580949; doi:10.5713/ab.25.0042)
Supplement: Supplementary file 2 [file ab-25-0042-supplementary-2.pdf]

Supplement 2. Primers used in qRT-PCR

Gene name	GenBank No.		Sequences (5' - 3')	Size (bp)
Beta actin (ACTB)	NM_173979.3	Forward	GGATGATGATATTGCTGCGCTC	195
		Reverse	GTCAGGATGCCTCTCTTGCTC	
18S	NR_036642.1	Forward	AGTACGCACGGCCGGTACAGT	130
		Reverse	CAGCGCCCGTCGGCATGTATT	
Interleukin 1 beta (lL-1β)	NM_174093.1	Forward	TTACTACAGTGACGAGAATGAGCT[illegible]	222
		Reverse	GCTGGATGTTTCCATCTCCCAT	
Claudin 1 (CLDN1)	NM_001001854.2	Forward	GTCTTTGGGGGCGTGATCTT	204
		Reverse	TTGTTTTCCGGGGACAGGAG	
Claudin 4 (CLDN4)	NM_001014391.2	Forward	CGTCATCCGCGACTTCTACA	185
		Reverse	TAGACGTAGTTGCTGGCTGG	
Claudin 7 (CLDN7)	NM_001040519.2	Forward	TGGGAGTGAGAGCAAAGCTG	217
		Reverse	GACCGAAGGCTTTTGCTTGG	
Occuldin (OCLN)	NM_001082433.2	Forward	AGGTGAATGGGTCACGGAGG	217
		Reverse	ACATGGCTGGTGTCAGTGGT	
Zonula occludens-1 (ZO-1)	DAA17567.a	Forward	CCGTCCTCTTCCTGCTTGAC	103
		Reverse	TCACCCACATCGGATTCTACG	
